# Procedural Pediatric Sedation by Nurses: Available, Competent, and Safe

**DOI:** 10.1155/2012/820209

**Published:** 2012-04-10

**Authors:** Laetiscia Lavoie, Catherine Vezina, Emilie Paul-Savoie, Claude Cyr, Sylvie Lafrenaye

**Affiliations:** ^1^Department of Pediatrics, Faculty of Medicine and Health Sciences, Université de Sherbrooke, 3001, 12 Avenue Nord, Sherbrooke, QC, Canada J1H 5N4; ^2^Department of Hemato-Oncology, McGill University Health Centre, Montreal, QC, Canada H3H 2R9

## Abstract

Sedation and/or analgesia are standard of care for pediatric patients during painful intervention or medical imaging requiring immobility. Physician availability is frequently insufficient to allow for all procedural sedation. A nurse-led sedation program was created at the Centre Hospitalier Universitaire de Sherbrooke (CHUS) to address this problem. *Objective*. To evaluate the effectiveness and the safety of our program. *Methods*. A retrospective study of all the procedural sedations done over one year was performed. Complications were separated in four categories: (1) major complications (call for help; unexpected admission, aspiration, and code); (2) reportable sedation events (oxygen saturation <90%, bradycardia (more than 2 SD below normal for the age of the child), and hypotension (more than 2 SD below normal for the age of the child); (3) difficult sedation (agitation, inadequate sedation, and failure to perform the procedure), (4) minor complications. *Results*. 448 patients, 249 boys and 199 girls; received sedation for 555 procedures. Overall, 78% (432) of interventions were successfully accomplished: 0% of major complications, 8% of reportable sedation events; 5% of difficult sedation; 9% of minor complications. *Conclusion*. Our nurse-led sedation program compares favorably to other similar systems.

## 1. Introduction

Sedation and/or analgesia are standard of care for pediatric patients during painful intervention or medical imaging requiring immobility. Pharmacological sedation forms part of pediatric care in order to improve reliability of results or facilitate the procedure for the physician and the patient [1]. Procedural sedation is usually administered by anesthetists, physicians, intensivists, or emergency physicians [2]. Pediatric sedation requires particular attention and training, but availability of pediatric anesthesia is not always sufficient. As a solution, a nurse-led pediatric sedation program was developed in our center. This team is composed of qualified nurses under the supervision of pediatric-intensive care specialists. This team can provide a wide range of procedural sedations, including imaging, endoscopies, and punctures.

Great Ormond Street Hospital (GOSH) offers a similar program [3]. In this center, only sedations for magnetic resonance imaging (MRI) were administered by nurses. Concerns were raised by anesthetists about the safety of such a program [4] which then led to a number of recommendations that were published about procedural sedation in children [2]. These guidelines were aimed mainly at anesthetists [5–7]. We used these recommendations to establish a first version approved by our nurses and physicians. Our protocol called “sedation program for pediatric patients” for pediatric nurses was published within our hospital [8]. While it has been demonstrated that well-structured guidelines are important for caregivers to achieve secure care [2], these guidelines must be explicit about drugs and their dosage in order to be used appropriately according to procedure, weight, and age of the child. Briefly, guidelines are based upon a list of complications and potential adverse effects and relate to solutions on how to manage these impediments. Our protocol respects all safety norms. In particular, during interventions, a nurse, assigned individually to each child, is monitoring basic vital signs, physiologic parameters, and the presence of any adverse effects.

Given that our nurses are not restricted to only one procedure, but cover multiple kinds of procedural sedation, the main goal of this retrospective study was to evaluate if our nurse-led program is efficient and safe.

## 2. Method

To evaluate the safety of this program, a retrospective study of all children who received procedural sedation in our institution (CHUS) over an entire year was performed. A complete review of different important characteristics for sedation was made: previous sedation, procedures, drugs used, disease, and complications.

These complications were classified in four categories: (1) major complications (call for help; unexpected admission, aspiration, code); (2) reportable sedation events (oxygen saturation <90%, bradycardia (more than 2 SD below normal for the age of the child), and hypotension (more than 2 SD below normal for the age of the child); (3) difficult sedation (agitation, inadequate sedation, and failure to perform the procedure); (4) minor complications. Nausea and vomiting were not reported in the sedation flow sheet and, as such, no data are available.

A variety of procedures were performed by the nurses as allowed by our program. These procedures were classified as painful or nonpainful. Painless procedures solely requiring immobility, such as medical imaging (MRI, CT, fluoroscopy), and electroencephalography (EEG) were done without opiates. Painful procedures such as endoscopy (gastric, colonic, and bronchoscopic), bone marrow aspiration, gastrostomy, central line insertion, painful imaging (such as guided biopsy), and minor orthopedic procedures were done using multimodal therapy including opiates or ketamine. Sedation for manometry, cystography, and urodynamic studies was also performed using our protocol. Strict criteria were used by nurses before any sedation such as, ASA that had to be less than 3, no active upper respiratory tract infection, no acute neurological condition, no high fever, and no snoring or sleep apnoea. If those conditions were present, the case had to be discussed beforehand with the intensivist on duty for the sedation unit.

### 2.1. Comparison with Other Centers

A comparison with other published results from pediatric sedation programs was done. A Medline search using the following words, pediatric sedation, nurse-led, procedural sedation was performed to find these programs. We also did a manual search of bibliographies to find relevant articles. We compared the CHUS program with two program types: those administered by (1) anesthetists, physicians, intensivists, or emergency physicians and (2) nurses.

### 2.2. Statistical Analysis

Usual descriptive statistics were made. Nonparametric test (Mann-Whitney) and *χ* ^2^ were used to determine difference between groups. A logistic regression was used to identify independent risk factors for major complication. The program StatView was used. 

## 3. Results

**Figure 1:**
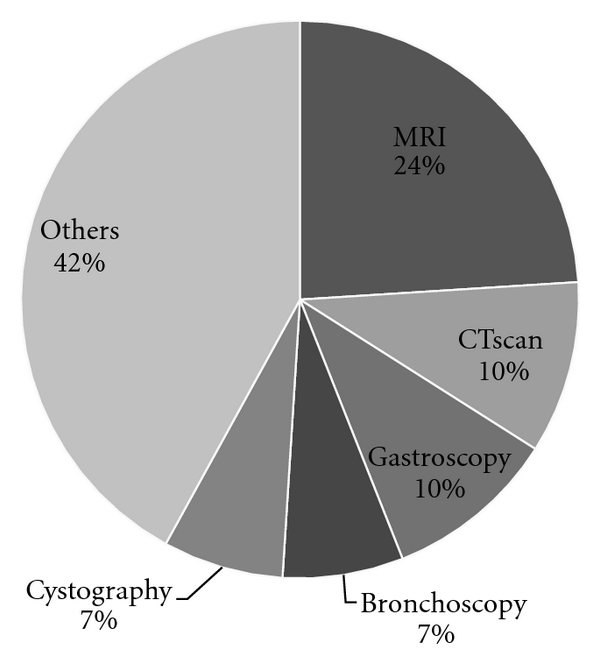
Most frequent procedures.

A total of 555 procedures were performed in 448 children. Some children had more than one procedural sedation over our study year. The age varied between 3 weeks and 18 years old, with an average of 4 years and 10 months. The majority of patients were boys (57%) (Table 1). More than 40 different procedures under sedation were done (Figure 1) and were grouped within 6 categories. Overall, 49% of the procedures done were painful and 51% were not. MRI was the most frequent with 24%, followed by the Computed Tomography scan (CT) 10%, gastroscopy (10%), bronchoscopy (7%), and cystography (7%). Seventy-four children underwent more than one intervention, with one child receiving seven sedations at different times during the year. Fifty-four percent of children had received sedation before. The majority of these previous sedations (91%) were done without complication.

Almost 50% of children presented a chronic medical condition at the time of the procedure. The most important were asthma (7%), development delay (5%), and neonatal leukemia (4%). Seventeen percent had a disease in the weeks before intervention including otitis media (4%), pneumonia, and upper airway tract infection (2%).

### 3.1. Drugs Used

The most frequent agent used was midazolam in 65% of procedures. Other sedatives used were ketamine (22%), fentanyl (21%), chloral hydrate (8%), and pentobarbital (8%). Fifty-two percent of children received a combination of 2 drugs, the most frequent being midazolam/ketamine and midazolam/fentanyl.

### 3.2. Complications

Overall, 78% of attempted procedural sedation was successfully done and was accomplished without any complications. We had a 22% rate of complication that consisted of major complications (0%), reportable sedation events (8%), difficult sedation (5% with 2.3% of failure), and minor complications (9%). No child had any long-lasting sequelae because of a sedation complication.

### 3.3. Risk Factors Associated with Complications

Reportable sedation events were associated with the use of fentanyl, endoscopies such as bronchoscopy and gastroscopy, and smaller weight (but not age) and with recent or chronic diseases. With the use of lorazepam or of more than one drug, chronic disease and endoscopy were associated with difficult sedation (Table 2). It is possible to quantify the impact of these risk factors on complications. In fact, reportable sedation events were more frequent in endoscopies (adjusted odds ratio (AOR) 2,2 (95% CI; 1,1–4,6), in children with chronic disease AOR 2,0 (95% CI; 1,0–3,8) and with the use of fentanyl AOR 3,4 (95% CI; 1,6–7.0).

### 3.4. Comparison with Other Programs

Once our complications were reviewed, we compared our findings to other institutions also using sedation protocols. The populations compared are similar to ours. The children have the same range of age. We can also notice that we carry out sedation in the same contexts, predominantly medical imaging procedures, but also a wide variety of other procedures (Figure 1). Another interesting point is to compare the sedation procedure with the ASA distribution, but this data was rarely available for the other studies reported. In our cohort everyone had an ASA classification of less than 3 (as by our protocol, no ASA > 3 are allowed to be done by nurses). Because no consensus exists on the definition of a procedural complication, we provide the complication rate in our institution for the type of complications described in each study (2). We believe these values are comparable given the similarities between the populations (Table 3).

Some of these programs are led by physicians (anaesthetists, intensivists) and others by nurses. One difference among these professionals is that anaesthetists are trained to perform anaesthesia and sedation. They are therefore the most qualified clinicians to accomplish procedural sedation, but not enough of them are available. Their clinical sedation outcomes are frequently reviewed. The study by Cravero (2006), which is a prospective observational study of a multispecialty group of sedation providers (anaesthesiologist, physician, and nurse), including 30,000 files of children who had received a sedation, reported oxygen desaturation with supplementation oxygen need in 157 cases out of 10,000 (1.57%) and an incidence of apnea in 0.42% of cases [9]. Cravero published another prospective study in 2009 and found similar results, showing the consistency of work performed by anaesthetists [10].

Two nurse-led sedation programs were found in the literature. In these programs, only MRI sedations were performed. In the study by the GOSH team, they found a 5% failure rate and reported that 33 children (33%) needed oxygen supplementation [3]. The second group from Minnesota University, using pediatric sedation since 1991, initially obtained inadequate sedation in 8.2% of patients. After 4 years of practice, this percentage fell to 3% [9]. Both these programs permitted a significant increase in the number of pediatric imaging.

Complication numbers fluctuate according to different centers and years. In 1997, Malviya reviewed 1,500 files with a 20% rate of complications. Of this number, 13% consisted of inadequate sedation, 5.5% of oxygen desaturation, and 3.4% of sedation failure [1]. In 2009, another study reported 1.15% of sedation failure [11]. Some studies have been done in specific populations. For example, Haque studied complications after sedation in pediatric oncology. In this case, the group found a 2.4% rate of oxygen desaturation and 0.6% rate of apnea [12]. 

## 4. Discussion

### 4.1. Program Efficiency

The main objective of this retrospective study was to evaluate if the CHUS nurse-led program was comparable to other programs mainly on the aspect of safety (rate of unexpected complications). We found an overall success rate of 78% with 13% significant complications. Five percent of our procedural sedation was not completed on the basis of sedation failure or because of a reportable sedation event. These results are similar to the literature [1]. Difficulties of comparing programs and studies reside in the fact that there is no standardized definition for pediatric complications, and authors have their own interpretation biases. We adjusted for this by comparing only similar complications. A nurse-led service at GOSH had a 3% failure rate during MRI sedation against 2.3% for our cohort. A sedation review done by nonanesthetists, published in 1997, obtained a 20% complication rate and 13% difficult sedations.

### 4.2. Risk Factors

 We observed several factors that were associated with complications in procedural sedation. These factors can be subdivided as follows: (1) factors related to the child and (2) factors related to the procedure.

Factors that relate to the child include the presence of chronic disease. This was observed in 50% of the study population. In these children, diagnostic procedures are often numerous and painful. Unfortunately, these risks factors are difficult to avoid. Also one consideration is the child's weight. Our findings show that low weight but not age is associated with a higher risk of reportable sedation events. This could be explained by the fact that sicker children often have a lower weight, and more frequent diagnostic procedures need to be performed. These children may also have a more unpredictable metabolism.

The second group of risk factors, related to the procedure, includes the drugs used, the combination of medications, and the procedures performed. Fentanyl was associated with more reportable sedation events. Fentanyl is an opiate used for moderate and deep sedation [8]. It is used in combination with benzodiazepines for painful procedures such as endoscopies and lumbar punctures. On the other hand, endoscopies are the interventions that lead to more complications; this could be explained by the fact that this drug combination is preferred in this type of intervention because of the pain and time involved. Often the longest procedure will require more drugs, and hence, increasing the complication rate of these procedures. Using more than one drug was also associated with difficult sedation. Multiple medications are used because they have synergistic effects. However, it is also possible that a difficult sedation (e.g., profound anxiety or increased amount of pain) will require more than one drug, even if not scheduled at the beginning. The last factor significantly associated with major complications and difficult sedation is the type of endoscopy. These procedures are intrusive and can cause a lot of anxiety.

Our analysis identifies risk factors to consider during procedural sedation. For example, 50% of children in our cohort had a chronic disease. This was statistically associated with reportable sedation events (*P* < 0.05). Similar results were found for endoscopic interventions (broncho-, gastro-, colono-) (*P* < 0.001) and the use of fentanyl (*P* < 0.001). As a result of our findings with respect to respiratory complications during bronchoscopy, we now perform the bronchoscopies in the PICU for children less than six months of age.

## 5. Conclusion

The evaluation of 555 files of pediatric patients who received sedation in the CHUS nurse-led program showed a comparable safety to other programs found in the literature despite the greater variety of procedural sedation offered by our program compared to GOSH and Minnesota University teams. This study also identified a list of important risk factors to consider when it is time to choose sedation or to require a general anesthesia.

We demonstrated that the efficacy and safety of our nurse-led sedation program are very similar to other comparable programs [3, 9]. However, the range of procedures offered by our program is much larger. Indeed, all pediatric procedures requiring analgesia, anxiolysis, and immobility are performed by our nurses. This allows more sedations to be performed at our institution. By demonstrating the comparable rate of reportable sedation event, rate of inadequate sedation, and failure rates of this program, we hope it will be used in other hospitals, resulting in a greater proportion of children eligible for sedation.

## Figures and Tables

**Table 1:** Population characteristics.

Characteristics	Percentage (%)
Male	57

Presence of a chronic disease	50
Asthma	7
Development delay	5
Neonatal leukemia	4

Presence of an acute disease	17
Otitis media	4
Pneumonia and upper airway tract infection	2

Have a previous sedation	54
Complication to previous sedation (any kind)	9

Drug used	
Midazolam	65
Ketamine	22
Fentanyl	21
Chloral hydrate	8
Pentobarbital	8

**Table 2:** Association test between risk factors: *P* values were calculated using *χ* ^2^ analysis.

Risk factor	Reportable sedation event *n* = 46 (%)	Other: minor complication, difficult sedation, no complication *n* = 509 (%)	*P* value	Difficult sedation *n* = 28 (%)	Adequate sedation *n* = 527 (%)	*P* value
Fentanyl (*n* = 118)	24 (52)	94 (18)	<.0001	4 (14)	114 (22)	0.3546
Lorazepam (*n* = 37)	2 (4)	35 (7)	0.0507	4 (14)	33 (6)	0.0085
Endoscopy (*n* = 120)	23 (50)	97 (19)	<.0001	1 (4)	119 (23)	0.0173
Age (month)	54 ± 57	59 ± 51	0.5757	60 ± 44	58 ± 52	0.8471
Weight (kg)	17 ± 15	20 ± 15	0.0208	21 ± 14	19 ± 15	0.2436
Chronic disease (*n* = 270)	30 (65)	240 (47)	0.0189	14 (50)	256 (49)	0.0220
Recent disease (*n* = 92)	13 (28)	79 (15)	0.0261	2 (7)	90 (17)	0.1683

**Table 3:** Comparison of complications in different studies on pediatric procedural sedation.

Studies	*n*	Procedures	Mean age ± SD (yr) (min–max)	Described complication	% found in each study	Equivalent % in our cohort
Sury et al., 1999 [3]	1155	MRI	N/A	Failure	5	2.3

Beebe et al., 2000 [9]	572	MRI	5 ± 4 (2 mo–14 yo)	Inadequate sedation	7.9	7.3

				Total	20.1	22

Malviya et al., 1997 [1]	1140	MRI (48%) CT (27%) Cardiac (22%)	3 ± 3.7	Inadequate sedation Failure Desaturation ≤90%	13.2 3.78 5.5	8 2.3 5.77

				Total	6.52	22

Lightdale et al., 2009 [11]	5045	Imaging (81%)	3.3 (1.4–6.4)	Serious adverse events* Failure Desaturation^§^	1.92 1.17 0.57	0 2.3 0

Haque and Fadoo, 2010 [12]	499	Oncology	4.2 (6 mo–14 yo)	Desaturation^¥^ Apnea	2.4 0.6	5.77 0

Cravero et al., 2006 [13]	30037	Imaging (60%) Oncology (9%) GI (6%)	0–6 mo : 6% 6 mo–2 yo : 23% 2–8 yo : 47% 8 yo+ : 29%	Desaturation ≤90% Apnea	1.57 0.24	5.77 0

Cravero et al., 2009 [10]	49836	Imaging (60%) Oncology (14%) GI (11%)	0–6 mo : 2% 6–12 mo : 6% 1-2 yo : 12% 2–4 yo : 21% 4–8 yo : 28% 8 yo+ : 29%	Desaturation ≤90% for 30 s Apnea	1.54 5.75	5.77 0

Lavoie, 2012	448	Imaging (41%) GI (10%)	4.1 ± 4.3 (1 mo–18 yo)	Failure Desaturation ≤90% Apnea Major complication	2.3 5.77 0 0	— — — —

*Serious adverse effects define as allergic reaction, aspiration, cardiovascular complications, need for resuscitation, unplanned admission, use of reversal agents, abnormal SpO2, prolonged sedation, and paradoxical reaction.

^§^Desaturation defines as a sustained drop in oxygen saturation 5% from baseline for more than 1 minute and unresponsive to blow-by oxygen at 6 L/min, and/or head repositioning, suctioning, or stimulation.

^¥^Transient desaturation which was improved by head repositioning and increasing oxygen flow.
